# College and Hospital Notes

**Published:** 1907-10

**Authors:** 


### COLLEGE AND HOSPITAL NOTES.

---

Dr. Roswell Park, professor of surgery at the University of Buffalo, will hold surgical clinics at the Buffalo General Hospital three times a week,—namely, Tuesdays, Thursdays and Saturdays during the sixty-second annual session of the medical department.

---

Harold Dickenson, Senior, M.B., F.R.C.S., at present an associate in anatomy at the Wistar Institute of Anatomy, Philadelphia, has been elected to the chair of anatomy and as director of the anatomical laboratory at Syracuse University, to take the place formerly held by Dr. George M. Price. Dr. Senior was educated in England at Durham University. He was first assistant demonstrator of anatomy at Durham University, then junior demonstrator of anatomy at Charing Cross Hospital Medical School, London. He came to Canada and engaged in the practice of medicine successfully for a number of years. His love for investigation in the field of anatomy led him to give up practice. He went to The Medico-Chirurgical College as demonstrator of anatomy but gave up that position to devote himself to investigation and became an associate in anatomy at Wistar Institute. His first published paper was on The Anatomy of the Heart of the Tarpon. He is now about to publish a treatise on the Anatomy of the Shad. He will have supervision of the departments of histology and embryology, and will secure an able assistant, trained in laboratory work to take the place of Professor Reese, who has accepted a position in The University of West Virginia.

---

Gustave M. Meyer, B.S., Sc.D., for four years assistant to the chair of physiological chemistry at Columbia University, New York, has been elected to the chair of physiological chemistry in the Medical College of Syracuse University. Dr. Meyer received his degree of Sc.D., at the University of Geneva, Switzerland.

His thesis was entitled, The Influence of Repeated Hemorrhages on the General Composition of the Blood. He has published many papers giving the results of his investigations, the latest of which will appear in the October number of the American Journal of Physiology, under the title, The Elimination of Radium in Normal and in Nephrectomised Animals. He will succeed Dr. George A. Hanford, who resigns to enter business.

---

Frank P. Knowlton, A.M., M.D., at present associate professor of physiology at Syracuse University has been advanced to the head of the department of physiology. A competent laboratory assistant to the chair of physiology will be selected at the earliest possible moment and, probably, will be ready for duty at the opening of college.

---

The plans of McKim, Meade & White for the new Children's Hospital at Rockaway Beach have just been submitted to the Association for improving the condition of the poor, which raised $250,000 toward the construction of this seaside institution for tuberculous children. The plans show a series of three-story pavilions, surrounded by broad verandas, with accommodations in each building for sixty patients. The pavilions, fourteen in number, are placed parallel to each other and at right angles to the ocean, except the two pavilions at either end of the series which run parallel to the ocean. As a site for the hospital, the city has agreed to reserve 1000 feet of its new five-mile beach at Rockaway. According to the Committee on Tuberculosis of the Charity Organisation Society, the new hospital is the result of the successful work carried on by its sister organisation for the past three years at its Sea Breeze hospital at Coney Island. Fresh air and outdoor life combined with careful medical oversight have worked such marked changes to the majority of the 110 little cripples that have been cared for at Sea Breeze, that this experimental station soon attracted wide attention throughout the country and finally led the Board of Estimate and Apportionment to set aside the stretch of beach that will provide amply for the hospital wards, the kindergartens and school rooms, the nurses training school and the pathological laboratory and all the necessities and conveniences which appertain to a modern hospital, sanatorium, and school which are called for by the final plans of the new Seaside Hospital. Among American cities, New York will then have the first seaside hospital for the treatment of bone tuberculosis in children, as it already has the first municipal country sanatorium for consumptives, and as it has long had the most efficient system for the administrative control of this disease.

